# *Porphyromonas gingivalis* affects neutrophil pro-inflammatory activities

**DOI:** 10.3389/fcell.2025.1419651

**Published:** 2025-01-28

**Authors:** Agnieszka Zimny, Alicja Płonczyńska, Wiktor Jakubowski, Natalia Zubrzycka, Jan Potempa, Maja Sochalska

**Affiliations:** ^1^ Department of Microbiology, Faculty of Biochemistry, Biophysics and Biotechnology, Jagiellonian University, Krakow, Poland; ^2^ Doctoral School of Exact and Natural Sciences, Jagiellonian University, Krakow, Poland; ^3^ Department of Oral Immunity and Infectious Diseases, University of Louisville School of Dentistry, Louisville, KY, United States

**Keywords:** porphyromonas gingivalis, gingipains, Bcl-2 family proteins, apoptosis, neutrophils, macrophages, periodontitis

## Abstract

Porphyromonas gingivalis is the primary pathogen responsible for the development of periodontal inflammatory disease. Although gingipains are the major virulence factor of the pathogen, their role in impairing apoptosis and immune cell function is not fully understood. To investigate the impact of gingipains on neutrophil viability and function, we conducted studies using murine HoxB8 neutrophils and primary human neutrophils infected with wild-type strains of *Porphyromonas gingivalis* (W83 and ATCC 33277), or a gingipains-null mutant with deleted gingipains encoding genes, or wild-type bacteria preincubated with specific gingipain inhibitors. Flow cytometry revealed that wild-type *Porphyromonas gingivalis* had a marked effect on neutrophil viability regulated by anti-apoptotic proteins belonging to the Bcl-2 family; however, these effects were independent of gingipain expression or activity. Importantly, experiments using primary human neutrophils and macrophages revealed that gingipains play a significant role in the disruption of immune cell functions via the induction of reactive oxygen species and inactivation of neutrophil elastase activity. Additionally, although gingipains played a role in modulating the IL-8-dependent inflammatory response of human neutrophils, they did not affect the expression levels of pro-inflammatory cytokines TNF-α and IL-6.

## 1 Introduction

Gingipains are cysteine proteases that function as the major virulence factors of *Porphyromonas gingivalis* by regulating both viability and the pathogenic potential necessary for disease development (Guo et al., 2010). There are two arginine-specific gingipains, RgpA and RgpB, and one lysine-specific gingipain, Kgp; that hydrolyze Arg-Xaa and Lys-Xaa peptide bonds, respectively (Guo et al., 2010; Xu et al., 2020). Although both arginine-specific gingipains contain an identical catalytic domain, RgpA also contains hemagglutinin-adhesin domains (similar to those in Kgp) (Guo et al., 2010; Seers et al., 2006; Li and Collyer, 2011). Gingipains, which account for 85% of the proteolytic activity of *Porphyromonas gingivalis* (Guo et al., 2010; Potempa et al., 1997), are located on the surface of the outer membrane of the bacterial envelope or released into the medium in soluble form (Guo et al., 2010; Xu et al., 2020). They play a crucial role at every stage of infection, contributing to tissue damage and impairment of host innate immune responses (Guo et al., 2010; Chen et al., 2023). During the initial phase of infection, gingipains RgpA and Kgp, bind to host extracellular matrix proteins via their hemagglutinin-adhesin domains to facilitate tissue colonization by pathogens (Guo et al., 2010; Pathirana et al., 2007; Pathirana et al., 2008). In addition, RgpA activates factors IX and X, as well as prothrombin (Guo et al., 2010; Imamura et al., 1997; Imamura et al., 2001a; Imamura et al., 2001b), whereas Kgp degrades fibrinogen and fibrin, thereby affecting clot formation and stability leading ultimately to increased bleeding and bacterial access to hemoglobin (Li and Collyer, 2011); however, it is their role in deregulating host defense mechanisms that has a direct effect on progression of inflammatory periodontal disease. To this end gingipains protect *Porphyromonas gingivalis* by, among other things, degrading cationic antibacterial peptides such as LL-37 and α-defensins, which are essential for bactericidal activity of neutrophils (Carlisle et al., 2009; Gutner et al., 2009). Furthermore, the pathogenic role of gingipains is dependent on both their concentration and the stage of infection. Lipopolysaccharide (LPS) derived from *Porphyromonas gingivalis* increases secretion of two variants of IL-8: IL-8_72aa_, which exerts strong chemotactic effects on neutrophils, and IL-8_77aa_, which requires limited proteolysis by gingipains for full activity (Dias et al., 2008; Sochalska and Potempa, 2017). During the initial phase of infection, gingipains reduce the activity of IL-8_72aa_ to impair neutrophil chemotaxis and enable bacterial colonization, while during the later phase of infection, they generate a hyperactive variant of IL-8_77aa_ (i.e., IL-8_69aa_) that promotes neutrophil migration to the site of infection (Sochalska and Potempa, 2017). Moreover, elevated levels of gingipains during the initial phase of infection lead to degradation of both soluble and membrane-bound forms of TNF-α, thereby impairing host inflammatory responses (Sochalska and Potempa, 2017; Mężyk-Kopeć et al., 2005).

Neutrophils (also known as polymorphonuclear leukocytes [PMNs]) play a key role in the innate immune response by maintaining tissue homeostasis, including that of the periodontium (Jiang et al., 2021). These cells eliminate bacteria through phagocytosis, discharge of cytotoxic granules, formation of neutrophil extracellular networks (NETs), production of reactive oxygen species (ROS), and secretion of pro-inflammatory cytokines (e.g., TNF-α, IL-8, IL-6, and IL-1β) (Sochalska and Potempa, 2017; Jiang et al., 2021; Bryzek et al., 2019; Guentsch et al., 2009; Ling et al., 2015). To evade the innate immune response, microorganisms have developed several virulence factors that can delay, enhance, or induce programmed cell death (Fox et al., 2010). Previous research shows that *Porphyromonas gingivalis* affects neutrophil responses by altering their viability and function, thereby promoting periodontitis (Sochalska and Potempa, 2017). In addition, LPS and lipid A derived from *Porphyromonas gingivalis* delay neutrophil apoptosis, leading to accumulation of these cells at the site of infection, thereby prolonging inflammation (Sochalska and Potempa, 2017; Murray and Wilton, 2003; Preshaw et al., 1999), while the TLR-4 inhibitor polymyxin B helps to suppress anti-apoptotic mechanisms (Sochalska et al., 2020). Apoptosis is regulated by proteins belonging to the Bcl-2 family, which includes both pro-apoptotic (e.g., Bak, Bax, and Bim) and anti-apoptotic (e.g., Bfl-1/A1, Mcl-1, and Bcl-xL) proteins (Kale et al., 2018). Mcl-1 is a key anti-apoptotic protein that extends neutrophil viability by sequestering the pro-apoptotic proteins Bak and Bax (Murphy and Caraher, 2015; Willis et al., 2005; Thomas et al., 2010). In turn, A1 protein levels in neutrophils are regulated primarily via the PI3K and JAK/STAT signaling pathways and linked to cell-mediated responses to microbial and pro-inflammatory stimuli (i.e., LPS and GM-CSF) (Vier et al., 2016). Furthermore, research shows that application of a selective inhibitor of the anti-apoptotic protein Bcl-xL (A-1331852) triggers neutrophil apoptosis in inflamed tissues (Carrington et al., 2021).

*Porphyromonas gingivalis* prolongs neutrophil viability regulated by anti-apoptotic proteins belonging to the Bcl-2 family (Sochalska et al., 2020; Prucsi et al., 2023); however, the role of gingipains remains unclear. The present study demonstrates that gingipains do not affect neutrophil or macrophage viability regulated by Bcl-2 family proteins, nor do they affect development of human neutrophil inflammatory responses mediated by TNF-α and IL-6. However, these proteases have a significant effect on ROS production, neutrophil elastase (NE) activity, and IL-8-dependent inflammatory responses by human neutrophils, thereby contributing to development and progression of periodontitis.

## 2 Materials and methods

### 2.1 HoxB8 cell lines and cell cultures

The *in vitro* HoxB8 model system was used for this study (as previously described (Sochalska et al., 2020)). Opti-MEM (Gibco, Waltham, MA, United States) supplemented with 10% FBS (Sigma-Aldrich, St. Louis, MO, United States), 250 μM L-glutamine (Gibco, Waltham, MA, United States), 100 U/mL penicillin and 100 μg/mL streptomycin (Life Technologies, Warszawa, Poland), 30 mM β-mercaptoethanol (Sigma-Aldrich, St. Louis, MO, United States), 2% Stem Cell Factor supernatant (SCF, obtained from the genetically modified CHO cell line (Wang et al., 2006)) and 1 µM β-estradiol (Sigma-Aldrich, St. Louis, MO, United States) was used to culture five independent lines of immortalized murine HoxB8 neutrophil progenitors (WT1-WT5). Differentiation of neutrophil progenitors into neutrophils was initiated by the removal of β-estradiol from the culture medium and continued for 4 days. Differentiated neutrophils were suspended in Opti-MEM medium supplemented with 2% FBS, 250 μM L-glutamine, 30 mM β-mercaptoethanol and 2% SCF supernatant.

### 2.2 Human neutrophil isolation

Neutrophils were isolated from human peripheral blood obtained from healthy volunteers at the Regional Blood Donation and Blood Treatment Center (Kraków, Poland). Neutrophils were isolated according to a previously described protocol (Bryzek et al., 2019) and suspended in RPMI medium (Gibco) supplemented with 2% FBS.

### 2.3 *Porphyromonas gingivalis* strains and bacterial culture

The wild-type strains of *Porphyromonas gingivalis* (fimbriae-free W83 and ATCC 33277) and the mutant strain devoid of gingipain activity in the *Porphyromonas gingivalis* W83 strain genetic background (∆KRAB) were used for the study. Bacteria were grown anaerobically at 37°C for 7 days on Brain Heart Infusion blood agar plates (BHI, Becton Dickinson, BD, Franklin Lakes, NJ, United States) with yeast extract (BioShop Canada Inc., Burlington, ON, Canada) supplemented with 0.5 mg/mL L-cysteine (BioShop Canada Inc., Burlington, ON, Canada), 10 μg/mL hemin (Sigma-Aldrich, St. Louis, MO, United States), 0.5 μg/mL vitamin K (Sigma-Aldrich, St. Louis, MO, United States) and, in the case of the mutant strain, 1 μg/mL tetracycline (BioShop Canada Inc., Burlington, ON, Canada) (as previously described (Prucsi et al., 2023)). On day −2, *Porphyromonas gingivalis* was anaerobically liquid-cultured using an enriched BHI medium. On day −1, the bacteria were washed with phosphate-buffered saline (PBS) and then suspended in fresh BHI medium at an optical density (OD)_600 nm_ = 0.1. On day 0 (experimental day), bacteria were washed with PBS and suspended in fresh PBS at an optical density (OD)_600 nm_ = 1 (corresponding to a concentration of 1 × 10^9^ Colony Forming Units (CFU) per 1 mL).

### 2.4 Neutrophil stimulations

Neutrophils were infected with *Porphyromonas gingivalis* strains and stimulated with *Porphyromonas gingivalis*-derived LPS Standard or polymyxin B (InvivoGen, San Diego, CA, United States; Cat. No. tlrl-pglps, tlrl-pmb, respectively). Depending on the condition, bacteria were treated with 1 μM of gingipain inhibitors KYT-1 and KYT-36 (Peptide Institute, Inc., Ibaraki-shi, Osaka, Japan).

After stimulation, supernatants were collected for ELISA and neutrophil elastase activity assay. Cells were harvested with cold PBS and, in the case of HoxB8 neutrophils, additionally treated with accutase (Sigma-Aldrich, St. Louis, MO, United States) for cytometric analysis and protein isolation.

### 2.5 Flow cytometry

To determine viability, cells were stained with Annexin V BV421 (BD Horizon, #563973, Franklin Lakes, NJ, United States) diluted 1:200 in 1x Annexin V Binding Buffer (Invitrogen, Carlsbad, CA, United States). The production of ROS by cells was measured using a 20 μM solution of 2′, 7′-dichlorofluorescein diacetate (DCFH-DA) (Sigma-Aldrich, #D6883, St. Louis, MO, United States) in PBS. Neutrophils were stained with DCFH-DA solution for 20 min at 37°C and then washed with cold PBS (as previously described (Prucsi et al., 2023)). To assess the level of membrane-bound human neutrophil elastase, cells were stained with a solution of PE-conjugated mouse anti-human NE antibodies (BD Pharmingen, # 568908, Franklin Lakes, NJ, United States) for 20 min at 4°C and then washed with cold PBS. Fluorescence was measured using LSR Fortessa flow cytometer (BD, Franklin Lakes, NJ, United States), and data were analyzed using FlowJo v10 Software (TreeStar, Ashland, OR, United States).

### 2.6 Enzyme-linked immunosorbent assay (ELISA)

The secretion of pro-inflammatory cytokines TNF-α, IL-6, and IL-8 was analyzed by ELISA according to the protocols attached to the commercial kits Mouse TNF-alpha DuoSet ELISA, Mouse IL-6 DuoSet ELISA (both from R&D Systems, Minneapolis, MN, United States), Human TNF-alpha Uncoated ELISA Kit, Human IL-6 Uncoated ELISA Kit (both from Invitrogen, Waltham, MA, United States), ELISA MAX Standard Set Human IL-8 (BioLegend, San Diego, CA, United States). Absorbance was measured using a Flex Station 3 microplate reader (Molecular Devices, San Jose, California, United States).

### 2.7 Immunoblotting

Protein isolation was carried out using CHAPS lysis buffer (Sigma-Aldrich, St. Louis, MO, United States). The total protein concentration in cell lysates was determined by the Bradford assay using Flex Station 3 (Molecular Devices, San Jose, California, United States). Protein identification by Western blotting was performed according to the previously described protocol (Prucsi et al., 2023). The separation of the proteins was carried out by polyacrylamide gel electrophoresis (SDS-PAGE) using a 4% stacking gel and a 12% separating gel. The proteins were then electrotransferred to an Immobilon®^−PSQ^ membrane (Merck-Millipore, Burlington, MA, United States). The membrane was blocked with 5% milk or 5% BSA (both from BioShop Canada Inc., Burlington, ON, Canada) in PBS containing 0.1% Tween20 (BioShop Canada Inc., Burlington, ON, Canada). Incubation with primary antibodies against A1 (kindly provided by Prof. Marco Herold, WEHI Institute, Melbourne, Australia), Mcl-1 (Rockland, 600–401–394, Pottstown, PA, United States), Bcl-xL (Cell Signaling Technology, #2764, Danvers, MA, United States) and GAPDH (Cell Signaling Technology, #2118, Danvers, MA, United States) was performed overnight at 4°C. The membrane was then washed with PBS-Tween20 and incubated at room temperature with anti-rat/anti-rabbit secondary antibodies (Cell Signaling Technology, #7077/#7074, Danvers, MA, United States) conjugated to horseradish peroxidase. After washing with PBS-Tween20, the membrane was incubated with Western blotting Pierce™ ECL substrate (Thermo Scientific™, Waltham, MA, United States) and imaged on Hyperfilm (Cytiva, Marlborough, MA, United States). Images were taken using the ChemiDoc™ MP imaging system (Bio-Rad, Hercules, California, United States).

### 2.8 Neutrophil elastase activity assay

Neutrophil elastase activity was assessed using a solution containing 10 mM chromogenic substrate for human neutrophil elastase p-nitroanilide N-methoxysuccinyl-Ala-Ala-Pro-Val in DMSO (both from Sigma-Aldrich, St. Louis, MO, United States), 0.5 M Tris-HCL (pH = 7) and dH_2_O at a ratio of 1:1:8, which was then added to the supernatants at a ratio of 1:1. Kinetic measurements were taken every 30 s for 30 min at 37°C using Flex Station 3 (Molecular Devices, San Jose, California, United States).

### 2.9 Statistical analysis

Statistical analysis was conducted using a One-sample *t-*test, One-sample Wilcoxon test, One-way ANOVA, Kruskal–Wallis test, Unpaired *t-test,* and Mann-Whitney test using GraphPad PRISM 9 Software. Statistically significant differences were considered for *p*-values <0.05.

## 3 Results

### 3.1 Gingipains are dispensable for *Porphyromonas gingivalis-*induced survival of murine HoxB8 neutrophils

To determine the effect of gingipains on the viability and function of neutrophils, we conducted experiments using two cell models. One was based on an immortalized murine HoxB8 neutrophil progenitor cell line, and the other on primary human neutrophils, which were stimulated with *Porphyromonas gingivalis* wild-type strains W83 and ATCC 33277, respectively. In both models, neutrophils were infected with a triple mutant devoid of gingipain encoding genes *∆kgp∆rgpA∆rgpB* (ΔKRAB) in the strain W83 genetic background. Additionally, we infected neutrophils in both models with *Porphyromonas gingivalis* pretreated with gingipain inhibitors KYT-1 and KYT-36 to eliminate their activity but preserve the proteolysis-independent functions of hemagglutinin/adhesion domains of gingipains. *Porphyromonas gingivalis*-derived LPS Standard was used as a positive control to elicit neutrophils’ immune response.

**FIGURE 1:**
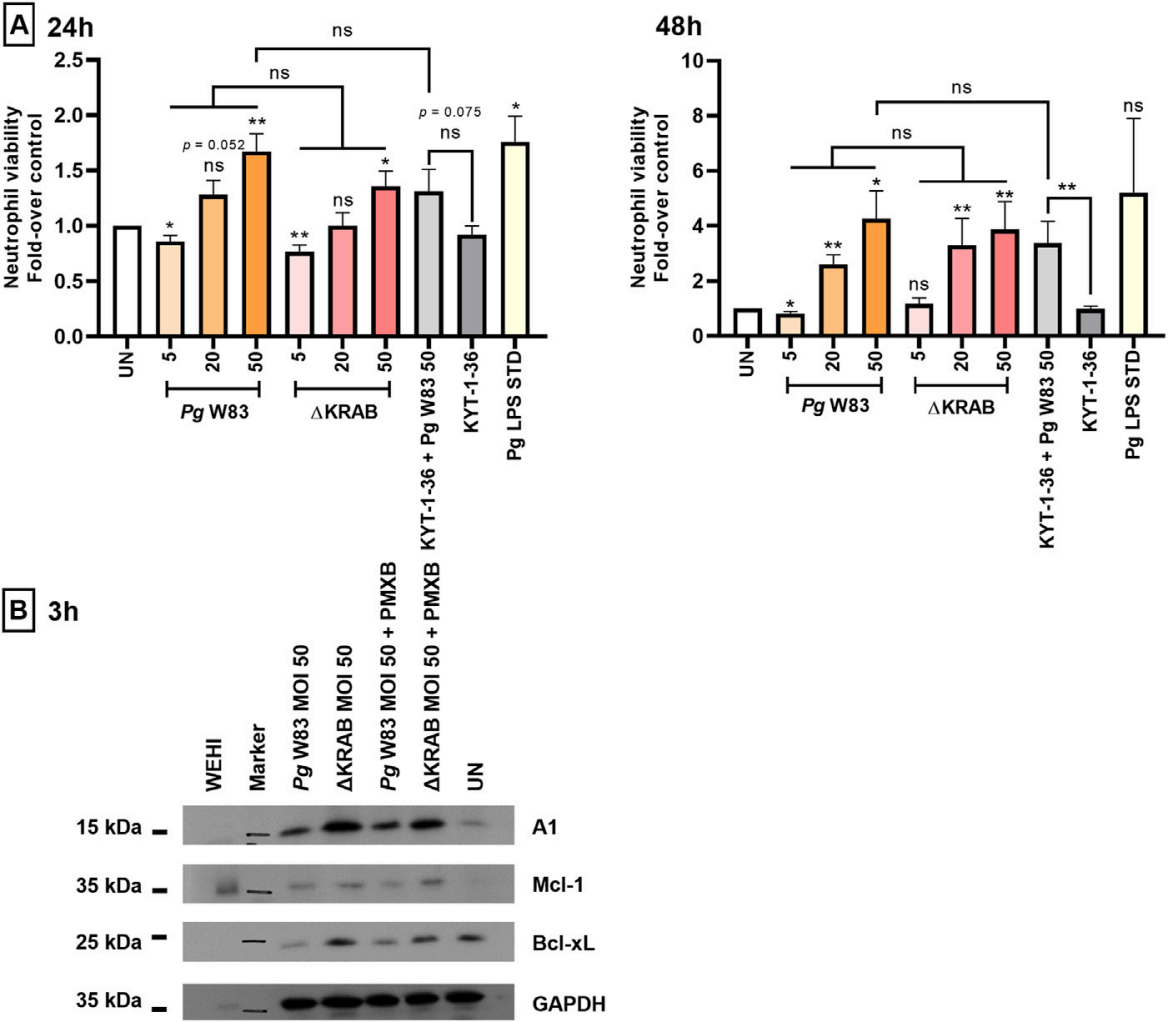
*Porphyromonas gingivalis* increases the viability of murine HoxB8 neutrophils via dose-dependent upregulation of anti-apoptotic proteins. **(A)** Murine HoxB8 neutrophils were stimulated with the WT-W83 or a mutant devoid of gingipain activity (∆KRAB) at an MOI 5, 20, or 50, and in the presence/absence of gingipain inhibitors KYT-1 and KYT-36 [both at 1 µM] or LPS inhibitor polymyxin B, PMXB [100 ng/mL]. A *Porphyromonas gingivalis*-derived LPS Standard (*Pg* LPS STD) served as a positive control [1 μg/mL]. Neutrophil viability at 24 and 48 h post-infection was analyzed by Annexin V BV421 staining followed by flow cytometry. Results are presented as the mean ± SEM (n = 3–12 independent experiments using the representative WT1-WT5 neutrophil lines) and expressed as a ratio between treated and untreated (UN) cells. Data are compared to control UN cells. *p < 0.05, **p < 0.01; ns, not significant (One sample *t*-test, One sample Wilcoxon test, One-way ANOVA followed by the Bonferroni post-hoc test, Kruskal–Wallis test followed by the Dunn post-hoc test, Unpaired *t*-test, and Mann-Whitney test). Non-significant difference of neutrophil viability after 48 h response of untreated cells (UN) and *Pg* LPS STD is due to large standard error and low numbers of repetitions (n = 3). **(B)** Western blotting results reveal expression of anti-apoptotic proteins belonging to the Bcl-2 family (A1, Mcl-1, and Bcl-xL) by murine HoxB8 neutrophils (one out of five WT cell lines) after 3 h of exposure to *Porphyromonas gingivalis*. Neutrophils were treated as described in **(A)**. In addition, cells were treated in the presence of polymyxin B (PMXB) to eliminate the cell response to LPS. WEHI-231(WEHI) cell lysate was used as the positive control.

Annexin V staining of murine HoxB8 neutrophils incubated with the wild-type strain of *Porphyromonas gingivalis* W83 at the multiplicity of infection (MOI) of 20 and 50 revealed an increase in the viability for up to 48 h post-infection (Figure 1A; Supplementary Figure S1) when compared to the untreated control. Similarly, infection with the gingipain null mutant (ΔKRAB) also prolonged HoxB8 neutrophils viability at MOI 20 and 50 for up to 48 h post-infection. Remarkably, there was no difference in the protective effect exerted by ∆KRAB and the parental WT-W83 strain. On the other hand, neutrophils exposure to gingipain inhibitors KYT-1 and KYT-36 pretreated bacteria at MOI 50 significantly increased cell survival only at 48 h post-infection (Figure 1A; Supplementary Figure S1). Collectively, these results suggest that gingipains do not contribute significantly to *Porphyromonas gingivalis* infection-induced survival of murine HoxB8 neutrophils, which is apparently dependent on other virulence factors like LPS, which was used here as a positive control to elicit the inflammatory response.

The prolonged viability of murine HoxB8 neutrophils in the presence of *Porphyromonas gingivalis* (Figure 1A) prompted us to examine the expression of anti-apoptotic proteins belonging to the Bcl-2 family (Figure 1B). Western blotting at 3 h post-infection revealed that both the W83 and the gingipain-null mutant (ΔKRAB) induced expression of three anti-apoptotic proteins, A1, Mcl-1, and Bcl-xL, by murine HoxB8 neutrophils. The same effect was observed in the presence of gingipain inhibitors in neutrophils infected with *Porphyromonas gingivalis* pretreated with KYTs (data not shown) indicating that *Porphyromonas gingivalis* strongly upregulated expression of anti-apoptotic proteins; however, neither gingipains nor their activity contributes to expression of A1, Mcl-1 and Bcl-xL in infected neutrophils. Abrogation of LPS effect by polymyxin B did not affect the strength of anti-apoptotic response elicited by *Porphyromonas gingivalis* at the level of expression of pro-survival Bcl-2 family proteins.

### 3.2 Gingipains are not essential for either ROS production or TNF-α secretion by infected neutrophils

**FIGURE 2:**
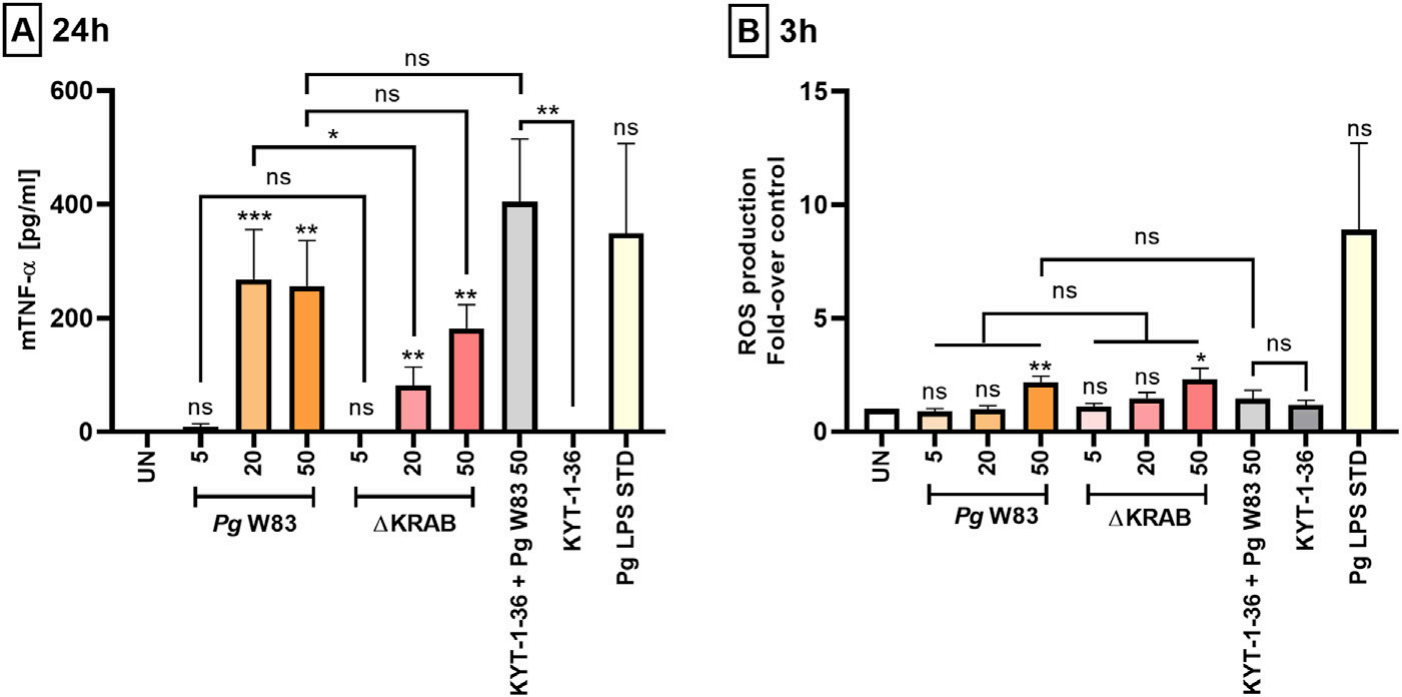
*Porphyromonas gingivalis* induces ROS production and affects the pro-inflammatory activity of murine HoxB8 neutrophils. (A) Production of TNF-α by murine HoxB8 neutrophils challenged with the WT-W83 strain and the ∆KRAB mutant in the absence and presence of gingipain inhibitors KYT-1 and KYT-36 [1 µM]. Cells were infected for 24 h with bacteria at MOI 5, 20, or 50, followed by measurement of TNF-α in the supernatants by ELISA. A *Porphyromonas gingivalis*-derived LPS Standard (*Pg* LPS STD) [1 μg/mL] was used as a positive control. Data are presented as the mean ± SEM of 4–11 independent experiments using the representative WT1-WT5 lines and are compared with data from untreated (UN) cells. *p < 0.05, **p < 0.01, ***p < 0.001, ns = not significant (One sample *t*-test, One sample Wilcoxon test, Kruskal–Wallis test followed by the Dunn post-hoc test, Unpaired *t*-test and Mann-Whitney test). TNF-α levels were measured in duplicate. **(B)** Production of ROS by murine HoxB8 neutrophils treated as in **(A)** was analyzed by flow cytometry after staining with DCFH_2_-DA for 3 h, followed by measurement of fluorescence emitted by oxidized DCF+. Results are presented as the mean ± SEM of 3–10 independent experiments calculated relative to the untreated (UN) cells (set arbitrarily at 1). *p < 0.05, **p < 0.01, ns = not significant (One sample *t*-test, One-way ANOVA followed by the Bonferroni post-hoc test and Unpaired *t*-test). Non-significant difference of TNF-α **(A)** and ROS **(B)** levels between untreated control (UN) and *Pg* LPS STD is due to large standard error and low numbers of repetitions (n = 3).

Periodontitis is accompanied by the host immune cells’ release of inflammatory mediators, which exacerbates periodontal tissue damage (Ramadan et al., 2020). Studies of neutrophils isolated from patients with chronic periodontitis report increased secretion of pro-inflammatory cytokines, i.e., IL-8, IL-6, TNF-α, and IL-1β (Ling et al., 2015). Here, we used a standard ELISA to examine the release of pro-inflammatory cytokine TNF-α by murine HoxB8 neutrophils in response to *Porphyromonas gingivalis* infection. The data showed that strain WT-W83 increased secretion of TNF-α by neutrophils at MOI 20 and 50, but not at 5 (Figure 2A). These observations indicate that induction of TNF-α release by neutrophils infected with *Porphyromonas gingivalis* occurs above a threshold level at MOI 5. Interestingly, neutrophils infected with ∆KRAB secreted measurable amounts of TNF-α but at MOI 20, they were at significantly lower levels than neutrophils infected with the parental strain (Figure 2A). This suggests that at MOI 50, the lack of gingipains was compensated for by other *Porphyromonas gingivalis* virulence factors in a manner independent of the gingipain activity as neutrophils infected with KYT-1 and KYT-36 treated bacteria secreted a large amount of TNF-α. Therefore, to test the impact of other *Porphyromonas gingivalis* virulence factors on TNF-α secretion, we employed two isogenic fimbriae mutants deficient in either Mfa1 or FimA (Supplementary Figure S2). Analysis of conditioned media of infected murine neutrophils revealed that the FimA protein was crucial for inflammatory response since cytokine production at MOI 20 and 50 was significantly reduced. In contrast, the Mfa1-deficient mutant did not elicit significant differences in TNF-α production compared to the ATCC 33277 strain.

Analysis of serum from patients with clinical attachment loss reports a correlation between elevated ROS levels and the levels of antibodies specific for three periodontal pathogens: *Porphyromonas gingivalis*, *Prevotella intermedia*, and *Eikenella corrodens* (Tamaki et al., 2014). In addition, increased ROS production by neutrophils was associated with the stage of periodontal disease (Aboodi et al., 2011). Analysis of the oxidative burst of murine HoxB8 neutrophils at 3 h post-infection revealed that *Porphyromonas gingivalis* strain W83 induced a generation of a significant amount of ROS compared to control only at MOI 50. A similar pattern was observed after infection with the gingipain-null mutant. Conversely, there was no significant ROS release by neutrophils in response to infection with *Porphyromonas gingivalis* with inhibited gingipain activity compared to neutrophils infected with the WT-W83 or ∆KRAB strains. This suggests that neither gingipains’ protein nor their activity is directly involved in activating the NADPH oxidase pathway in murine neutrophils (Figure 2B; Supplementary Figure S3). Of note, the *Porphyromonas gingivalis*-derived LPS Standard used as a positive control induced the measurable but statistically not significant production of ROS by neutrophils (Figure 2B).

### 3.3 Infection with the W83 and ATCC 33277 strains exerted an opposite, gingipain-independent effect on human neutrophil survival

Next, to verify the results obtained using the immortalized murine HoxB8 neutrophil model, we performed experiments using primary human neutrophils isolated from the peripheral blood of healthy donors. Using this model, we compared neutrophil responses triggered by infection with encapsulated, fimbriae-deficient strain W83 and fimbriated strain ATCC 33277 of *Porphyromonas gingivalis*. Notably, these two strains are commonly used in research on the role of *Porphyromonas gingivalis* in the development and progression of periodontitis but are seldomly compared back-to-back.

**FIGURE 3:**
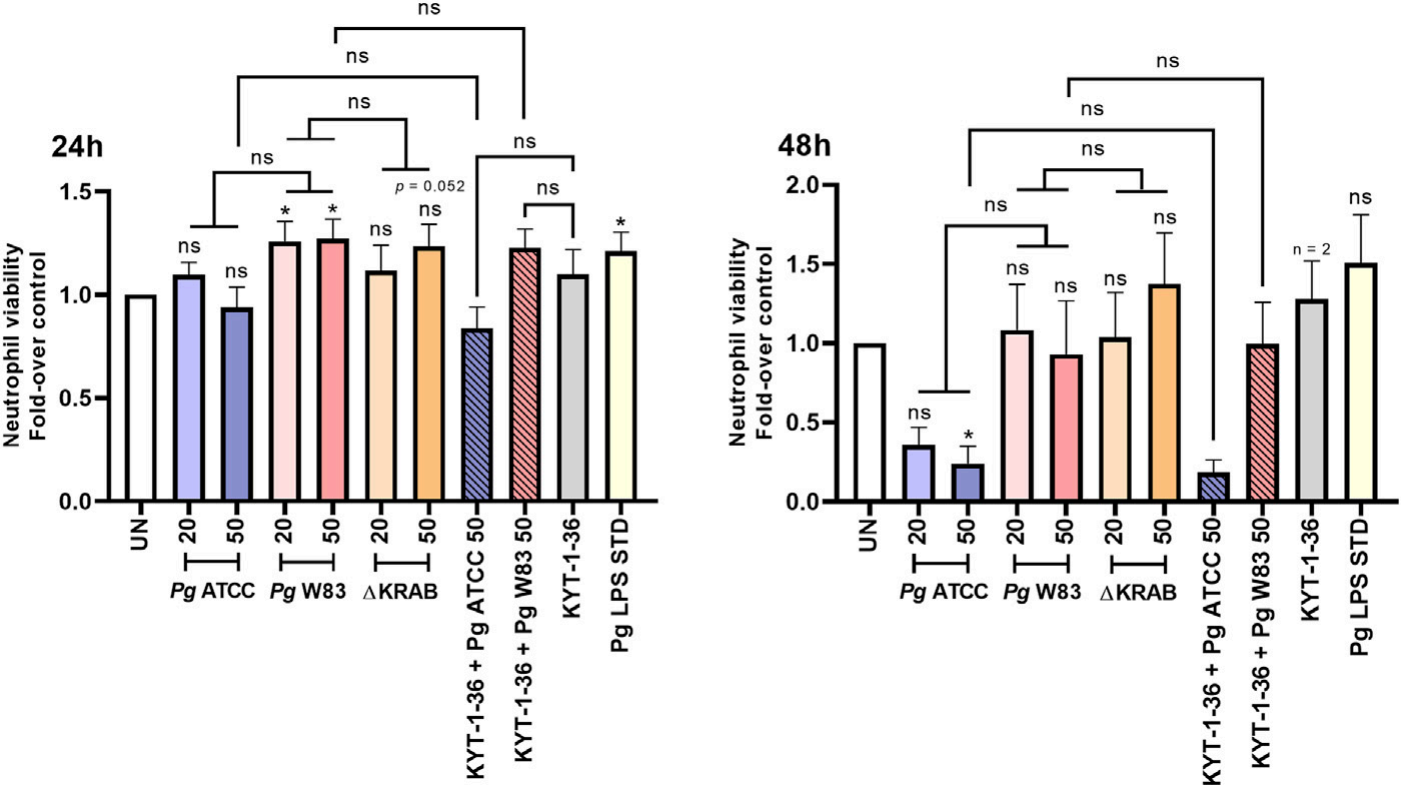
Infection with the W83 and ATCC 33277 strains exerted an opposite, gingipain-independent effect on human neutrophil survival. Primary human neutrophils were challenged with wild-type *Porphyromonas gingivalis* (W83 and ATCC 33277) or the mutant devoid of gingipain activity (∆KRAB) in the absence and presence of gingipain inhibitors KYT-1 and KYT-36 [1 μM]. Bacteria were used at MOI 20 and 50. A *Porphyromonas gingivalis*-derived LPS Standard (*Pg* LPS STD) served as a positive control [1 μg/mL]. Neutrophil viability at 24 and 48 h post-treatment was analyzed by flow cytometry after Annexin V BV421 staining. Data are presented as mean ± SEM of 2–11 independent experiments and expressed as a ratio between treated and untreated (UN) cells. Results are compared to control UN cells. *p < 0.05, ns = not significant (One sample *t*-test, One sample Wilcoxon test, One-way ANOVA followed by the Bonferroni post-hoc test, Kruskal–Wallis test followed by the Dunn post-hoc test and Unpaired *t*-test). Non-significant difference of neutrophil viability after 48 h response of untreated cells (UN) and *Pg* LPS STD is due to large standard error and low numbers of repetitions (n = 3).

Flow cytometry analysis revealed that the W83 at MOI 20 and 50 prolonged the viability of human neutrophils at 24 h post-infection, while the ATCC 33277 and gingipain-null mutant strain did not increase survival rates when compared to the untreated control. Interestingly, however, in stark contrast to the W83 strain, incubation of neutrophils with the ATCC 33277 strain for a prolonged time (48 h) resulted in a significant reduction in cell viability (Figure 3; Supplementary Figure S4). This indicates that infection with strains W83 and ATCC 33277 may affect human neutrophil survival differently. Additionally, and in line with the data from the murine neutrophil model, human neutrophils infected with ∆KRAB and *Porphyromonas gingivalis* with the gingipain activity quenched by KYTs did not show any significant difference in neutrophil viability at 24 or 48 h post-infection (Figure 3; Supplementary Figure S4), when compared to neutrophils challenged with W83. The results consistent with the observed response of mice neutrophils to *Porphyromonas gingivalis* infection strongly argue that gingipains do not affect neutrophil apoptosis. Of note, the response to treatment with *Porphyromonas gingivalis*-derived LPS significantly prolonged the survival of human (Figure 3) and mouse (Figure 1A) neutrophils in a similar manner.

### 3.4 Human neutrophil response to *Porphyromonas gingivalis* infection measured by cytokine secretion and ROS generation depends on the bacterial strain

**FIGURE 4:**
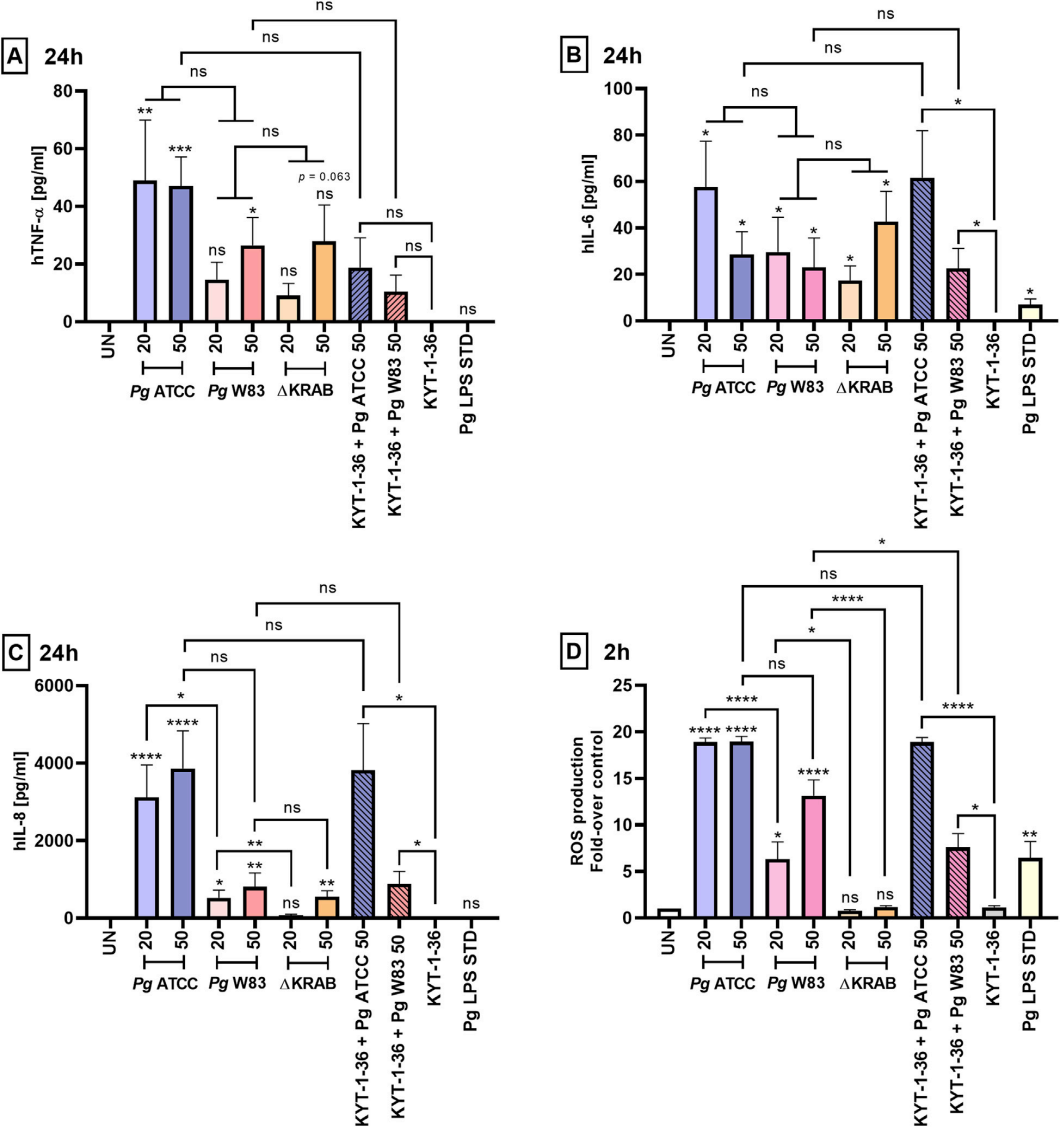
Gingipains induce strong inflammatory responses by primary human neutrophils. Production of **(A)** TNF-α, **(B)** IL-6, and **(C)** IL-8 by primary human neutrophils challenged for 24 h with the W83 and ATCC 33277 strains in the absence and presence of gingipain inhibitors KYT-1 and KYT-36 [1 μM], and with the mutant devoid of gingipains (∆KRAB). Bacteria were used at MOI 20 and 50, and cytokine levels in supernatants were measured by ELISA. A *Porphyromonas gingivalis*-derived LPS Standard (*Pg* LPS STD) served as a positive control [1 μg/mL]. Results are presented as the mean ± SEM of **(A)** 4–10, **(B)** 5–10, and **(C)** 4–9 independent experiments and compared with those from untreated (UN) cells. *p < 0.05, **p < 0.01, ***p < 0.001, ****p < 0.0001, ns = not significant (One sample *t*-test, One sample Wilcoxon test, Kruskal–Wallis test followed by the Dunn post-hoc test, Unpaired *t*-test and Mann-Whitney test). Pro-inflammatory cytokine levels were measured in duplicate. **(D)** Production of ROS by primary human neutrophils treated as in **(A, B, C)** was analyzed by staining with DCFH_2_-DA for 2 h, followed by measurement of fluorescence emitted by oxidized DCF+. Data are presented as the mean ± SEM of 3–9 independent experiments and expressed as a ratio between treated and untreated (UN) cells. *p < 0.05, **p < 0.01, ****p < 0.0001, ns = not significant (One sample *t*-test, One sample Wilcoxon test, One-way ANOVA followed by the Bonferroni post-hoc test and Unpaired *t*-test).

Proinflammatory cytokine secretion, oxidative burst, and degranulation represent immune responses of neutrophils to bacterial pathogens. Here we compared the response of human peripheral neutrophils to infection with two different strains of *Porphyromonas gingivalis* and evaluated the role of gingipains in the release of pro-inflammatory cytokines and generation of ROS. To this end, neutrophils were infected with *Porphyromonas gingivalis* ATCC 33277, W83, and gingipain-null W83 mutant strain (∆KRAB), and the level of secreted TNF-α, IL-6, and IL-8 in a conditioned media was determined at different time-points after infection. At 2 h post-infection with the ATCC 33277 strain, only IL-8 was detected (Supplementary Figure S5C). The highest levels of pro-inflammatory cytokines were found at 24 h post-infection with both, the W83 and ATCC 33277 strains (Figures 4A–C). In contrast to IL-6 secretion, which was induced to the same level regardless of *Porphyromonas gingivalis* strains, neutrophils infected with ATCC 33277 have a very strong tendency to secrete more IL-8 and TNF-α than neutrophils infected with W83. However, due to large standard deviations, the statistical significance of difference was reached only for IL-8 secretion induced by the *Porphyromonas gingivalis* strains at MOI 20. At 48 h post-infection, only the concentration of IL-8 was found at a significantly high level (>2,000 pg/mL) in the conditioned medium of neutrophils infected with the ATCC 33277 strain (Supplementary Figure S5C). Together, these findings suggest an additive effect related to the presence of gingipains and fimbriae in the latter strain. Notably, mice neutrophils and macrophages secreted much higher amounts of TNF-α in response to infection with *Porphyromonas gingivalis* than human neutrophils as indicated by the 10-fold higher concentration of the cytokine accumulated in the conditioned medium at 24 h (Figures 2A, 4A; Supplementary Figure S6).

To determine if gingipains play a role in the stimulation of cytokine secretion, neutrophils were infected with ∆KRAB or bacteria pretreated with gingipain inhibitors. The IL-8 concentration in a conditioned medium of neutrophils infected with ∆KRAB (24 h, MOI 20) was significantly reduced compared to neutrophils infected with the WT-W83 (Figure 4C). By contrast, the gingipains absence in the W83 ∆KRAB mutant did not affect the secretion of other cytokines (i.e., TNF-α and IL-6) after 24 h of infection (Figures 4A,B). Regardless of the *Porphyromonas gingivalis* strain, bacteria pretreatment with gingipain inhibitors did not affect cytokine release. Finally, we determined the response of human neutrophils to stimulation with *Porphyromonas gingivalis* LPS Standard.

In contrast to murine neutrophils (Figure 2A), we observed an increase only in IL-6 secretion after 24 h of stimulation with LPS. At the same time, the levels of TNF-α and IL-8 were comparable with those secreted by untreated control cells (Figures 4A–C). Cumulatively, it is remarkable that, except for a minor effect on IL-6 release, *Porphyromonas gingivalis* LPS Standard did not trigger other cytokines secretion in human neutrophils in stark contrast to murine HoxB8 cells.

To further investigate *Porphyromonas gingivalis* and its virulence factors on neutrophil function we measured the production of ROS by neutrophils. As in the case of cytokine secretion, at MOI 20, the ATCC 33277 strain stimulated the release of ROS at a significantly higher level from infected human neutrophils than W83. Remarkably, in contrast to murine neutrophils (Figure 2B), flow cytometry analysis ROS production revealed that gingipain proteins, as well as their activity, play an important role in inducing an oxidative burst by primary human neutrophils infected with *Porphyromonas gingivalis* W83. This is apparent from the finding that the production of ROS by neutrophils nearly totally ablated in the isogenic gingipain-null mutant ∆KRAB) and was significantly lower in cells infected with the W83 strain pretreated with gingipain inhibitors (KYT-1 and KYT-36). In contrast, inhibiting gingipain activity secreted by ATCC 33277 did not affect ROS generation by infected human neutrophils (Figure 4D; Supplementary Figure S7). Interestingly, in contrast to infection with the ATCC 33277 strain, ROS production by neutrophils exposed to the W83 was upregulated in an MOI-dependent manner. Exposure of neutrophils to the ATCC 33277 strain led to hyperactivation, resulting in increased generation of ROS. Notably, the effect persisted despite the elimination of gingipain activity by pretreatment of bacteria with the inhibitors. This suggests the involvement of fimbriae of the ATCC 33277 strain as well as lipopolysaccharide sialylation, in excessive neutrophil activation (Figure 4D; Supplementary Figure S7). As expected, the *Porphyromonas gingivalis*-derived LPS Standard activated ROS production but at a relatively low level. These results demonstrate that the *Porphyromonas gingivalis*-derived lipopolysaccharide Standard exerts similar effects on ROS generation by primary human neutrophils, and murine HoxB8 neutrophils (Figures 2B, 4D).

### 3.5 *Porphyromonas gingivalis* affects the level of neutrophil elastase on the neutrophil surface in a gingipain-activity-independent manner

**FIGURE 5:**
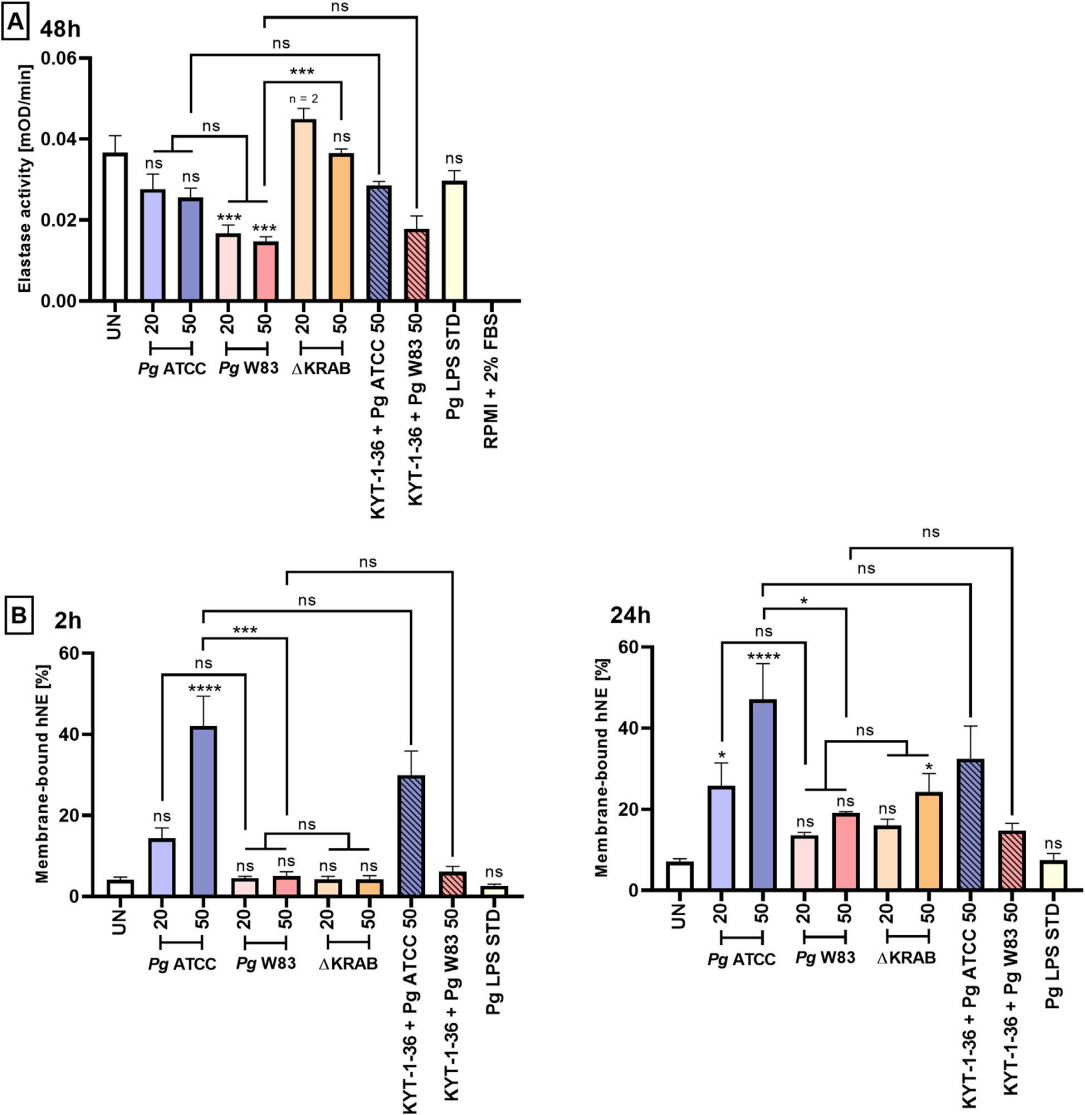
*Porphyromonas gingivalis* affects the level of neutrophil elastase on the neutrophil surface in a gingipain-activity-independent manner. (A) Primary human neutrophils were challenged with wild-type *Porphyromonas gingivalis* (W83 and ATCC 33277) or the mutant devoid of gingipain activity (∆KRAB) in the absence and presence of gingipain inhibitors KYT-1 and KYT-36 [1 μM], the supernatants were collected, and hNE activity was measured in the presence of chromogenic substrate after 48 h. Data are presented as the mean ± SEM of 2-3 independent experiments, and compared to UN cells. ***p < 0.001, ns = not significant (One-way ANOVA followed by the Dunnett post-hoc test, One-way ANOVA followed by the Bonferroni post-hoc test and Unpaired *t*-test). hNE activity levels were measured in duplicate. UN cells and cell culture medium alone (RPMI with 2% FBS) were used as negative controls for the experiments. **(B)** Primary human neutrophils were stimulated as in **(A)**, and membrane-bound hNE levels were analyzed by flow cytometry in the presence of PE-conjugated Mouse Anti-Human Neutrophil Elastase antibody after 2 and 24 h. Results (compared with UN cells) are presented as the mean +SEM of three independent experiments. *p < 0.05, ***p < 0.001, ****p < 0.0001, ns = not significant (One-way ANOVA followed by the Dunnett post-hoc test, One-way ANOVA followed by the Bonferroni post-hoc test and Unpaired *t*-test).

Finally, we assessed neutrophil degranulation triggered by interaction with *Porphyromonas gingivalis*. To this end, we challenged primary human neutrophils with different strains of *Porphyromonas gingivalis* and measured the NE activity in a conditioned medium. At 24 h post-infection, only ATCC 33277 caused an MOI-dependent increase of the NE activity, but changes were beyond the statistical significance. Compared to the parental W83 strain, the ∆KRAB mutant slightly increased the NE activity at MOI 20 after 24 h (Supplementary Figure S8). At 48 h, the NE activity was significantly reduced in condition media of neutrophils infected with W83, but not with the ATCC 33277 strain; in this case, a non-significand drop of activity was observed. Remarkably, the NE activity remained at the control level or slightly increased (MOI 20) when neutrophils were infected with ∆KRAB (Figure 5A). This result may suggest proteolytic inactivation of NE by gingipains. However, this conclusion is not supported by the lack of an effect of *Porphyromonas gingivalis* pretreatment with gingipain inhibitors prior to infection of neutrophils on the released NE activity. Of note, results obtained from the murine HoxB8 model did not reveal any significant difference in the NE activity at any time post-infection with the wild-type strain W83, ∆KRAB, or stimulation with *Porphyromonas gingivalis*-isolated LPS, or in the presence of gingipain inhibitors KYT-1 and KYT-36 (Supplementary Figure S9).

Since released elastase tends to be bound on the neutrophile surface (Bangalore and Travis, 1994), we finally asked if gingipains will affect membrane-bound human NE. Flow cytometry analysis confirmed that ATCC 33277 increased the level of membrane-bound NE at the higher *Porphyromonas gingivalis* dose (MOI 50). The increase was independent of the gingipain activity as bacteria pretreatment with gingipain inhibitors did not eliminate the effect. Furthermore, the effect was strain-dependent, as infection with strain W83 had no significant effect on the amount of NE on the cell surface regardless of the time point after infection (Figure 5B). Finally, the *Porphyromonas gingivalis*-derived LPS Standard did not affect either the activity of NE (Figure 5A; Supplementary Figure S8) or the level of membrane-associated NE (Figure 5B).

## 4 Discussion

Periodontitis is a chronic and multifactorial inflammatory disease caused by an imbalance between the subgingival microbiota and the host immune response (Liaw et al., 2022; Hajishengallis, 2014). The key pathogens involved in the progression of periodontitis are anaerobic Gram-negative bacteria belonging to the red complex, i.e., *Porphyromonas gingivalis*, *Tannerella forsythia,* and *Treponema denticola*, which are frequently accompanied by species from the orange complex (e.g., *Fusobacterium nucleatum* and *P. intermedia*) (Mohanty et al., 2019). The degree of disease progression and severity is closely related to the abundance of these pathogens in subgingival plaque (Mohanty et al., 2019). Although the red complex pathogens are considered to be the major etiological factor underlying the development of periodontitis, it is ultimately the host immune response (including hyperreactive neutrophils) that promotes the progression of the disease (Jiang et al., 2021; Hajishengallis, 2014; Matthews et al., 2007).

Gingipains, the primary virulence factors of *Porphyromonas gingivalis*, play a crucial role in manipulating the neutrophil response in the inflamed periodontium. Among host signaling pathways, these proteases target the TREM-1 receptor-dependent signaling. Arg-specific gingipains are responsible for the release of sTREM-1 (soluble TREM-1) from the neutrophil membrane to maintain chronic inflammation, while Lys-specific gingipains suppress inflammatory responses by degrading sTREM-1 (Sochalska and Potempa, 2017; Bostanci et al., 2013). Moreover, gingipains exert opposing effects on the complement system depending on their concentration, which reflects an abundance of *Porphyromonas gingivalis* in periodontal pockets. At low concentrations, gingipains activate complement components C3 and C5, resulting in the release of anaphylatoxins C3a and C5a, thereby promoting inflammation in the gingival tissue which provides essential nutrients for *Porphyromonas gingivalis* proliferation (Sochalska and Potempa, 2017; Potempa and Pike, 2009; Popadiak et al., 2007). At high concentrations, gingipains inactivate the complement system by degrading components C3 and C5, thereby preventing the elimination of pathogens by neutrophils (Sochalska and Potempa, 2017; Potempa and Pike, 2009; Popadiak et al., 2007).

In the physiological state, the consequence of the neutralization of pathogens is subsequent apoptosis of neutrophils followed by their efferocytosis by macrophages or dendritic cells (Leitch et al., 2008). Proper regulation of these processes is crucial for resolving inflammation and reducing damage to host tissues caused by hyperreactive neutrophils (Sochalska and Potempa, 2017). Our recently published *in vitro* studies confirmed that the effect of *Porphyromonas gingivalis* peptidyl arginine deiminase (PPAD) on delaying neutrophil-induced apoptosis is mediated by anti-apoptotic proteins belonging to the Bcl-2 family (Prucsi et al., 2023). In the present study, we show that gingipains do not affect the viability of murine or peripheral human neutrophils (Figures 1A, 3). Analysis of expression of the Bcl-2 family of anti-apoptotic proteins by murine HoxB8 neutrophils revealed that A1 protein levels were higher in neutrophils infected with the gingipain-null mutant (∆KRAB) than the wild-type W83. A similar effect was observed for the Mcl-1 and Bcl-xL proteins (Figure 1B), and in all cases, the effect on tested anti-apoptotic proteins was independent of MOI (data not shown). These results indicate that the expression of anti-apoptotic proteins in *Porphyromonas gingivalis*-infected neutrophils is not only gingipain independent but enhanced by the absence of the gingipain proteins or their activity. The increase in anti-apoptotic proteins is likely compensated by the expression of pro-apoptotic proteins such as Bim or Puma, which needs further investigation.

Disruption of the balance between neutrophil survival and apoptosis promoted by *Porphyromonas gingivalis* results in the accumulation of hyperactive neutrophils at the site of infection. Increased activation, which includes excessive ROS production, leads to progressive destruction of periodontal tissue, which in turn allows bacteria to access essential nutrients, thereby promoting pathogens’ survival and spread (Sochalska and Potempa, 2017; Zaric et al., 2010). Moreover, the oxidative burst in response to periodontal pathogens plays a key role in regulating cell signaling, including activation of the NF-κB and JNK pathways, which in turn affects the inflammatory response and induction of apoptosis (Liu et al., 2017). Our findings are in line with previously published data (Sochalska et al., 2020; Prucsi et al., 2023), and confirm that wild-type strain W83 and *Porphyromonas gingivalis*-derived LPS Standard induced ROS generation in both murine and human neutrophils (Figures 2B, 4D).

The present study showed a difference between the W83 and ATCC 33277 strains regarding ROS generation which was MOI-independent in the case of the latter strain. More differences were revealed by the application of gingipain inhibitors. Upon gingipain inhibition, the W83 strain generated lower levels of reactive oxygen species from infected neutrophils, while such reduction was not observed for the ATCC 33277 strain. The difference is likely due to the lack of fimbriae protein and the higher abundance of sialic acid residues on LPS in strain W83 that cumulatively weakens inflammatory responses to this strain (Zaric et al., 2017; Wielento et al., 2022) (Figure 4D).

The pathophysiology of periodontitis is associated with chronic inflammation propagated by pro-inflammatory cytokines released by host immune cells (Ling et al., 2015; Ramadan et al., 2020). TNF-α is an inflammatory mediator that may have both pro- and anti-apoptotic effects on neutrophils depending on its concentration (Noseykina et al., 2021; van den Berg et al., 2001). In turn, IL-6 is a pro-inflammatory cytokine that regulates neutrophil migration during inflammation (Hashizume et al., 2011; Fielding et al., 2008). The present study using the murine HoxB8 model showed that the wild-type W83 strain upregulates TNF-α release in an MOI-dependent manner after 24 h of infection (Figure 2A). In turn, infection of murine neutrophils with ∆KRAB affected TNF-α levels only at MOI 20 (Figure 2A). These observations suggest that at higher MOI, the lack of gingipains is most likely compensated for by the presence of other *Porphyromonas gingivalis* virulence factors. The gingipain-null mutant (∆KRAB) and its parental strain (W83) caused comparable secretion of TNF-α by human neutrophils after 24 h of infection, which correlates with the viability results (Figures 3, 4A). Similarly, ∆KRAB and strain W83 had comparable effects on the secretion of IL-6 by human neutrophils (Figure 4B). Of note, both *Porphyromonas gingivalis* wild-type strains induced the release of the same amount of pro-inflammatory cytokines TNF-α and IL-6 by human neutrophils (Figures 4A,B). Taken together, these data suggest that under tested conditions gingipains play no role in TNF-α and IL-6-mediated pro-inflammatory responses by human neutrophils but may affect the secretion of TNF-α by murine neutrophils depending on MOI. Importantly, gingipains’ activity depends strongly on pH, serum proteins, and redox status (Darveau et al., 2002; Seers et al., 2020; Rajapakse et al., 2002). Therefore, highly specific experimental approaches, which would take into account these gingipains’ vulnerabilities should be performed in the future.

IL-8 is a key chemokine involved in recruiting neutrophils to infected periodontal tissues (Finoti et al., 2017). Limited proteolysis of IL-8_77aa_ by gingipains results in the formation of a truncated hyperactive variant called IL-8_69aa_ (Sochalska and Potempa, 2017; Mikolajczyk-Pawlinska et al., 1998). Gingipains are also involved in degradation or activation of this cytokine, depending on their concentration, and the location and stage of infection. Our research revealed that gingipains increased secretion of IL-8 by human neutrophils in a MOI-dependent manner. Following infection with ∆KRAB at MOI 20, human neutrophils produced less IL-8 after 24 h than after infection with the W83 strain (Figure 4C). At a higher MOI, the lack of gingipain activity did not affect the secretion of IL-8 by human neutrophils (Figure 4C). Furthermore, at MOI 20, fimbriated ATCC 33277 induced IL-8 production at much higher levels than strain W83, indicating an additive effect related to the presence of gingipains and fimbriae (Figure 4C). Moreover, and in line with a previous study (Zaric et al., 2010), we found that a high level of IL-8 was associated with a low level of TNF-α, which promotes neutrophil survival and maintains chronic inflammation (Figures 4A,C).

NE, a serine protease found both in the primary granules of neutrophils and on the membrane of activated neutrophils, is responsible for eliminating microorganisms (Voynow and Shinbashi, 2021). Neutrophils from patients with periodontitis have higher NE activity than those from healthy donors (Guentsch et al., 2009). The present study using the ∆KRAB strain confirmed that gingipains, through proteolysis, reduced NE activity at the higher bacterial load (Figure 5A), but did not affect levels of membrane-bound NE (Figure 5B). In addition, we observed an inverse relationship between NE activity and neutrophil viability after 48 h of infection (Figures 3, 5A). In addition, strain W83 reduced NE activity to a greater extent than ATCC 33277, which markedly reduced neutrophil viability at 48 h post-infection (Figures 3, 5A). This relationship may be associated with the higher virulence of the W83 strain than that of ATCC 33277 in some infection models. Flow cytometry analysis also revealed that the level of membrane-bound NE in ATCC 33277 infected neutrophils was significantly higher than that of W83 infected cells, both 2 and 24 h postinfection (Figure 5B). Taken together, confirm a significant role of fimbriae of strain ATCC 33277 in the activation of inflammatory functions of neutrophils, measured not only in terms of ROS and pro-inflammatory cytokine production but also in terms of NE levels.

In conclusion, the present study demonstrates that infection with *Porphyromonas gingivalis* prolongs the survival of both murine and human neutrophils. Importantly, the data confirm that the prolonged viability of murine HoxB8 neutrophils is dependent on the induction of anti-apoptotic proteins belonging to the Bcl-2 family, i.e., A1, Mcl-1, and Bcl-xL. Furthermore, the present study provides strong evidence supporting the important role of gingipains in impairing neutrophil function, thereby promoting the development of periodontitis. The data confirm that gingipains upregulate IL-8 secretion by human neutrophils in an MOI-dependent manner during the late stage of *Porphyromonas gingivalis* infection. Moreover, gingipains strongly induce ROS production by primary human neutrophils, and decrease the activity of human NE significantly; this relationship is inversely proportional to neutrophil viability. Therefore, inhibition of gingipains and anti-apoptotic proteins belonging to the Bcl-2 family may be a promising strategy for the treatment of inflammatory periodontal disease.

## Data Availability

The raw data supporting the conclusions of this article will be made available by the authors, without undue reservation.
