# Correction: *Porphyromonas gingivalis* affects neutrophil pro-inflammatory activities

**DOI:** 10.3389/fcell.2025.1652545

**Published:** 2025-07-15

**Authors:** Agnieszka Zimny, Alicja Plonczynska, Wiktor Jakubowski, Natalia Zubrzycka, Jan Potempa, Maja Sochalska

**Affiliations:** ^1^Department of Microbiology, Faculty of Biochemistry, Biophysics and Biotechnology, Jagiellonian University, Krakow, Poland; ^2^Doctoral School of Exact and Natural Sciences, Jagiellonian University, Krakow, Poland; ^3^Department of Oral Immunity and Infectious Diseases, University of Louisville School of Dentistry, Louisville, KY, United States

**Keywords:** *porphyromonas gingivalis*, gingipains, Bcl-2 family proteins, apoptosis, neutrophils, macrophages, periodontitis

In the published article, there was an error in the **Funding** statement. This previously stated:

“The author(s) declare that financial support was received for the research and/or publication of this article. MS was supported by grants from the Foundation for Polish Science (FIRST TEAM program co-financed by the European Union under the European Regional Development Fund; grant number First Team/2017-4/40, POIR.04.04.00-00-42FE/17) and from the National Science Center, Poland (UMO-2020/39/D/NZ5/02075).”

The correct **Funding** statement appears below.

