# Supplementary material for: Porphyromonas gingivalis affects neutrophil pro-inflammatory activities
Source: Front Cell Dev Biol. 2025 Jan 28;13:1419651. doi: 10.3389/fcell.2025.1419651 (PMC11811088; doi:10.3389/fcell.2025.1419651)
Supplement: Supplementary file 1 [file DataSheet1.docx]

Supplementary Material

# Supplementary Figures


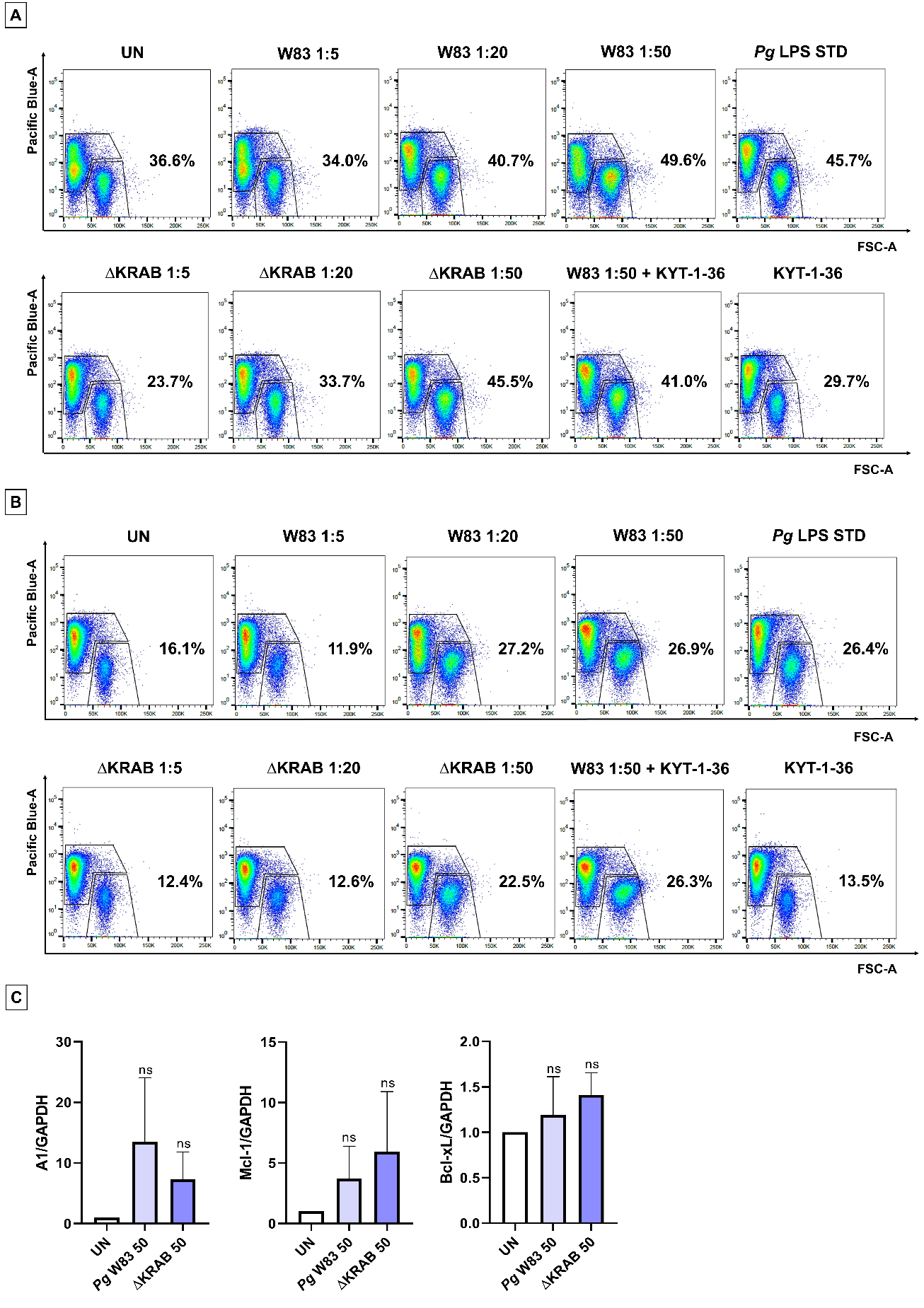
**Supplementary Figure S1. *P. gingivalis* increases the viability of murine HoxB8 neutrophils.** Murine HoxB8 neutrophils (a representative WT1 neutrophil line) were stimulated with the wild-type strain of *P. gingivalis* (W83) or a mutant devoid of gingipains (∆KRAB) at MOI 5, 20, or 50, and in the presence/absence of gingipain inhibitors KYT-1 and KYT-36 [both at 1 µM]. A *P. gingivalis*-derived LPS Standard (*Pg* LPS STD) served as a positive control [1 µg/ml]. Untreated (UN) cells were used as a negative control. Neutrophil viability at **(A)** 24 and **(B)** 48 h post-infection was analyzed by Annexin V BV421 staining followed by flow cytometry. Percentages refer to the Annexin V-negative population (lower gate). **(C)** Densitometry analysis of relative protein expression of anti-apoptotic proteins (A1, Mcl-1, and Bcl-xL) by murine HoxB8 neutrophils after 3 h of incubation with the wild-type strain of *P. gingivalis* (W83) or a mutant devoid of gingipains (∆KRAB) at an MOI 50. Results are presented as the mean ± SEM (n = 3 independent experiments using the three different HoxB8 lines). GAPDH was used as a control. Data are compared to control UN cells. ns, not significant (One sample t-test and One sample Wilcoxon test).


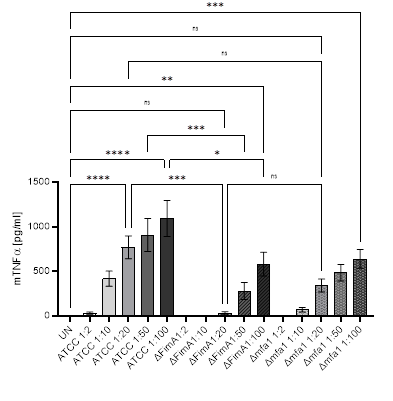


**Supplementary Figure S2.** **The** **FimA protein is crucial for HoxB8 neutrophils inflammatory response.** Wild-type HoxB8 neutrophils were either left untreated or stimulated for 3 h, with *P. gingivalis* wild-type strains ATCC 33277 and two derived mutants lacking fimbriae proteins *Δmfa1* and *ΔfimA* at MOI 2, 10, 20, 50, and 100. After the indicated time points, supernatants were collected. Production of murine TNF-α was analyzed in supernatants by ELISA. Quantification of results from five independent wild-type cell lines; bars show means ± SEM. Differences between groups were calculated by One-way ANOVA followed by Brown-Forsythe test; *p ≤ 0.05, **p ≤ 0.01, ***p ≤ 0.001, ****p ≤ 0.0001.

**
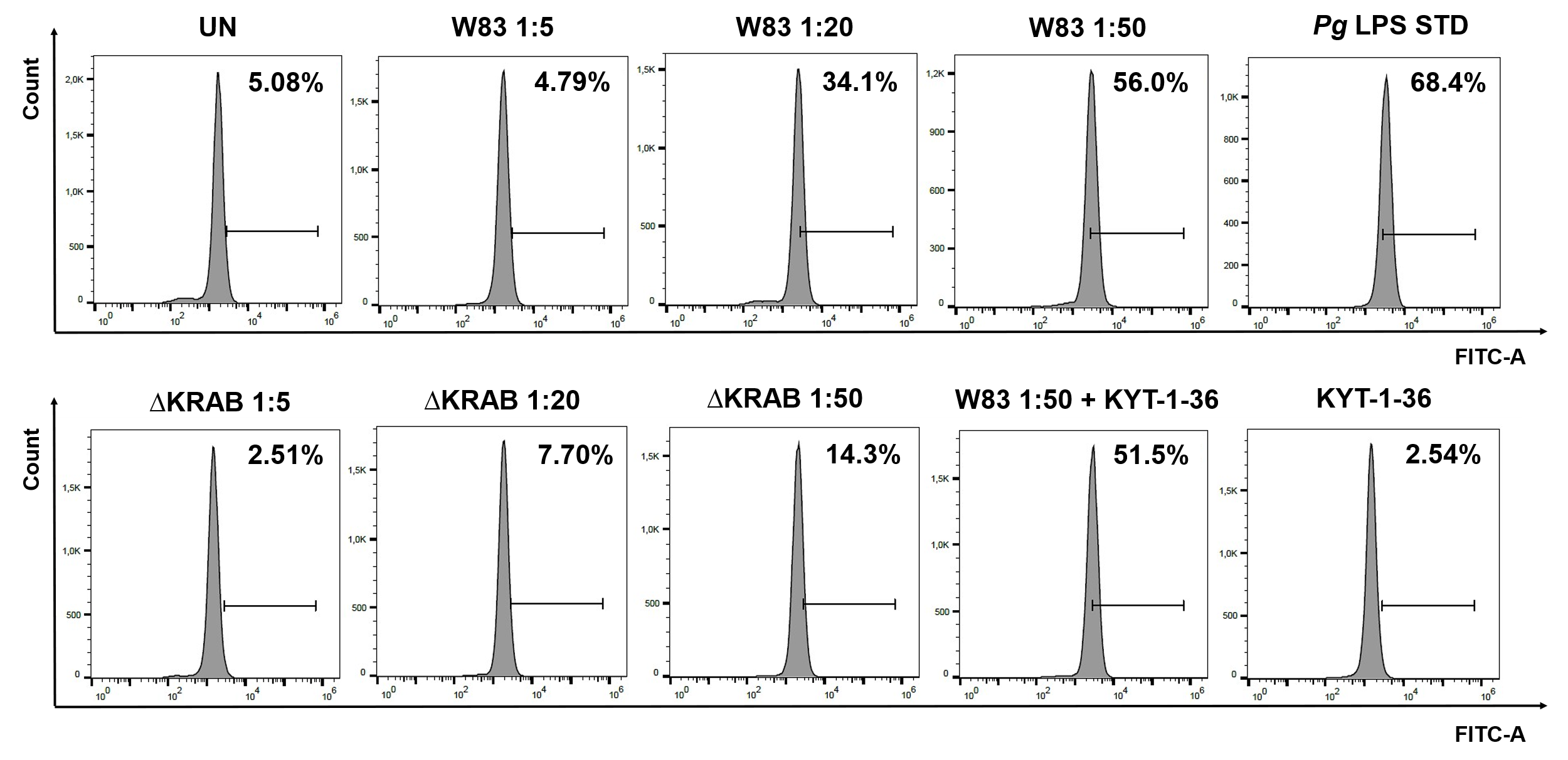
**

**Supplementary Figure S3. *P. gingivalis* induces ROS production by murine HoxB8 neutrophils.** Murine HoxB8 neutrophils (representative WT1 neutrophil line) were treated with the WT-W83 or the gingipain mutant at MOI 5, 20, and 50, where indicated gingipain inhibitors KYT-1 and KYT-36 were added [both at 1 µM]. *P. gingivalis*-derived LPS Standard (*Pg* LPS STD) served as a positive control [1 µg/ml]. Untreated (UN) cells were used as a negative control. Production of ROS by murine HoxB8 neutrophils was analyzed by flow cytometry after staining with DCFH_2_-DA for 3 h, followed by measurement of fluorescence emitted by oxidized DCF+.

**
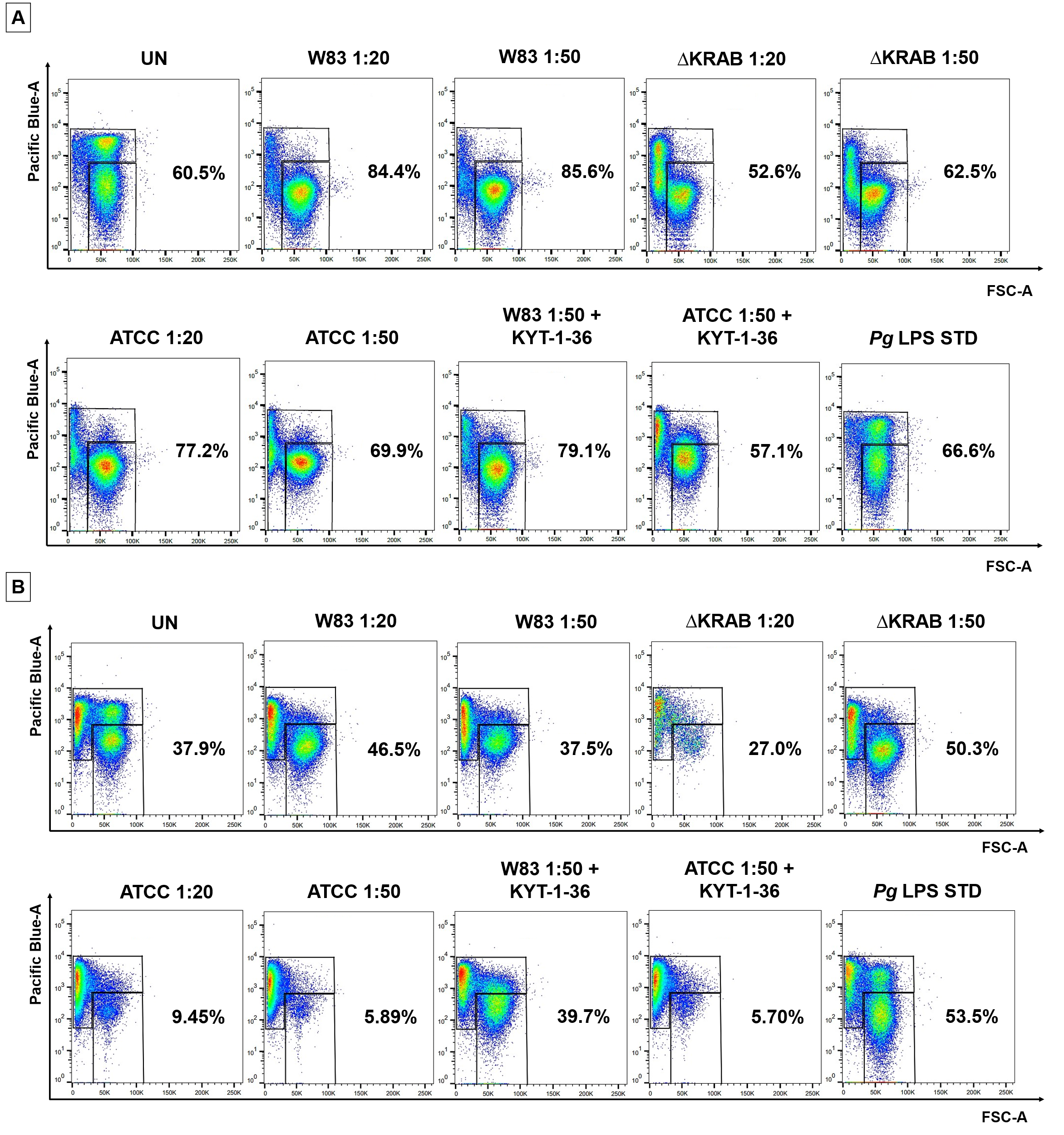
**

**Supplementary Figure S4. *P. gingivalis* prolongs peripheral human neutrophil survival up to 24 h of infection.** Primary human neutrophils were challenged with wild-type *P. gingivalis* (W83 and ATCC 33277) or the ∆KRAB mutant in the absence and presence of gingipain inhibitors KYT-1 and KYT-36 [both at 1 μM]. Bacteria were used at MOI of 20 and 50. A *P. gingivalis*-derived LPS Standard (*Pg* LPS STD) served as a positive control [1 µg/ml]. Untreated (UN) cells were used as a negative control. Neutrophil viability at **(A)** 24 and **(B)** 48 h post-treatment was analyzed by flow cytometry after Annexin V BV421 staining. Percentages refer to the Annexin V-negative population (lower gate).

**
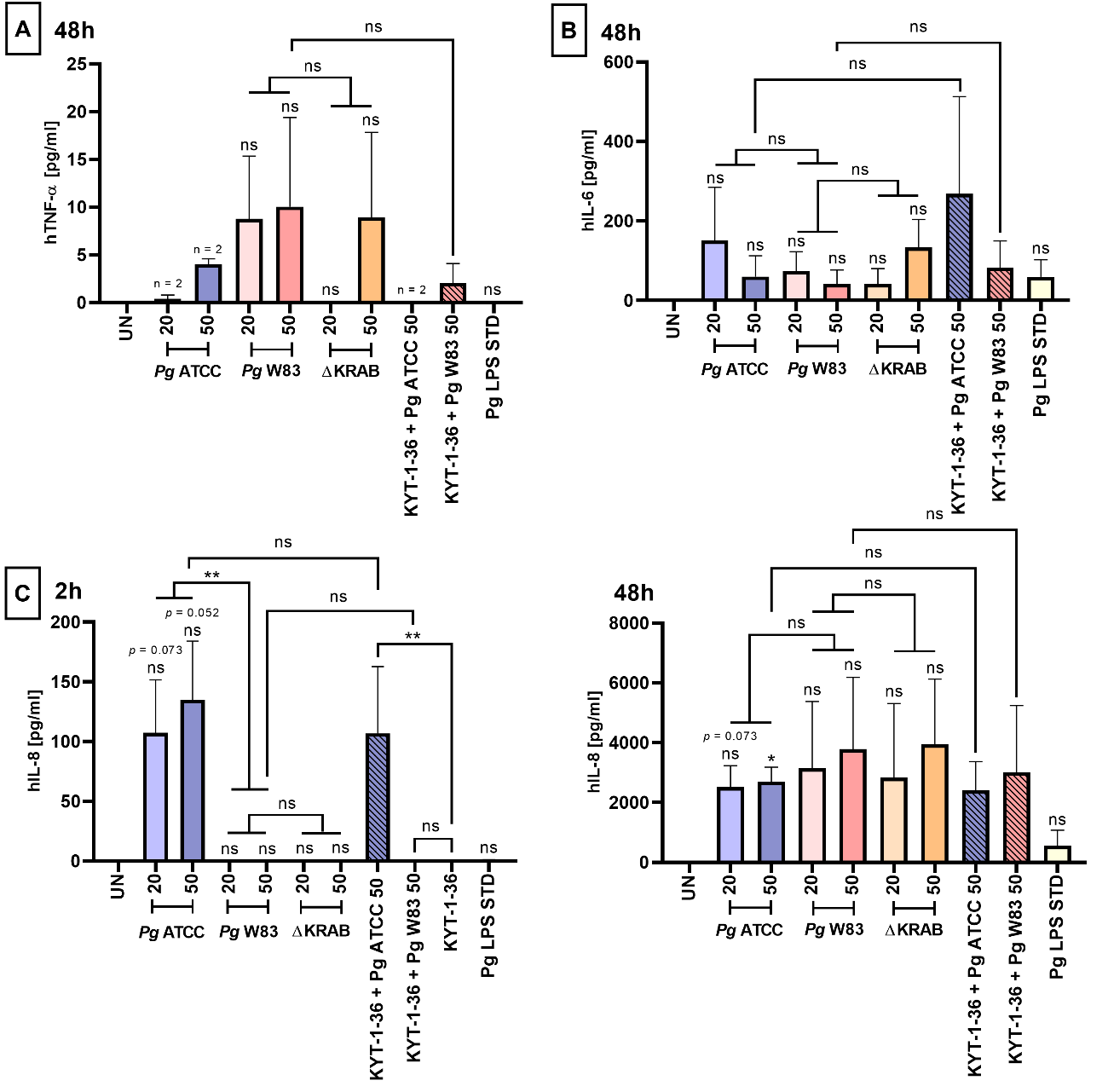
**

**Supplementary Figure S5. *P. gingivalis* induces pro-inflammatory activity of primary human neutrophils.** Production of **(A)** TNF-α, **(B)** IL-6, and **(C)** IL-8 by primary human neutrophils challenged with the WT-W83 or WT-ATCC 33277, and with the ∆KRAB mutant for **(A, B, C)** 48 and **(C)** 2 h in the absence and presence of gingipain inhibitors KYT-1 and KYT-36 [both at 1 μM]. Bacteria were used at MOI 20 and 50, and cytokine levels in supernatants were measured by ELISA. A *P. gingivalis*-derived LPS Standard (*Pg* LPS STD) served as a positive control [1 µg/ml]. Results are presented as the mean ± SEM of **(A)** 2-4, **(B)** 3-4, and **(C)** 3-9 independent experiments and compared with those from untreated (UN) cells. *p < 0.05, **p < 0.01, ns = not significant (One sample *t*-test, One sample Wilcoxon test, Kruskal-Wallis test followed by the Dunn post-hoc test, Unpaired *t*-test and Mann-Whitney test). Pro-inflammatory cytokine levels were measured in duplicate.

**
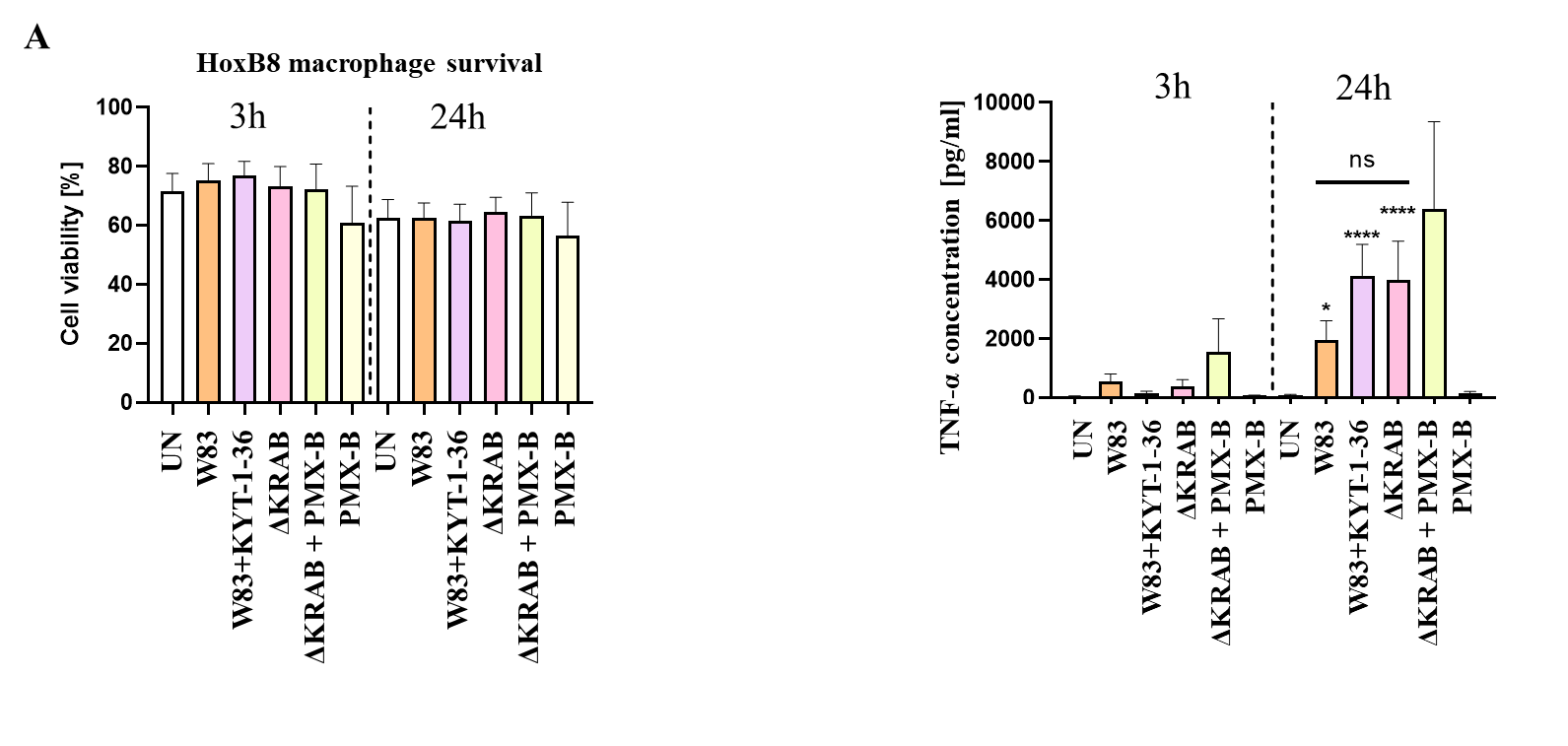
**

**Supplementary Figure S6. *P. gingivalis* induces secretion of TNF-α by macrophages, independently of cell survival.** Murine HoxB8 macrophages were either left untreated (UN) or stimulated with the WT-W83 or a gingipains-mutant (∆KRAB) at MOI 20, and in the presence/absence of gingipain inhibitors KYT-1 and KYT-36 [both at 2 µM] or LPS inactivator, polymyxin B [10 µg/ml] (PMX-B, Invivogen). **(A)** Macrophage survival at 3 and 24 h post-infection was analyzed by Annexin V-BV421 and propidium iodide (PI) staining followed by flow cytometry. **(B)** TNF-α concentration from medium collected at 3 and 24 h post-infection was analyzed by ELISA assay. Results are presented as the mean ± SEM (n = 10 independent experiments using three wild-type HoxB8 cell lines). *p < 0.05, ****p < 0.0001; ns, not significant (One-way ANOVA).


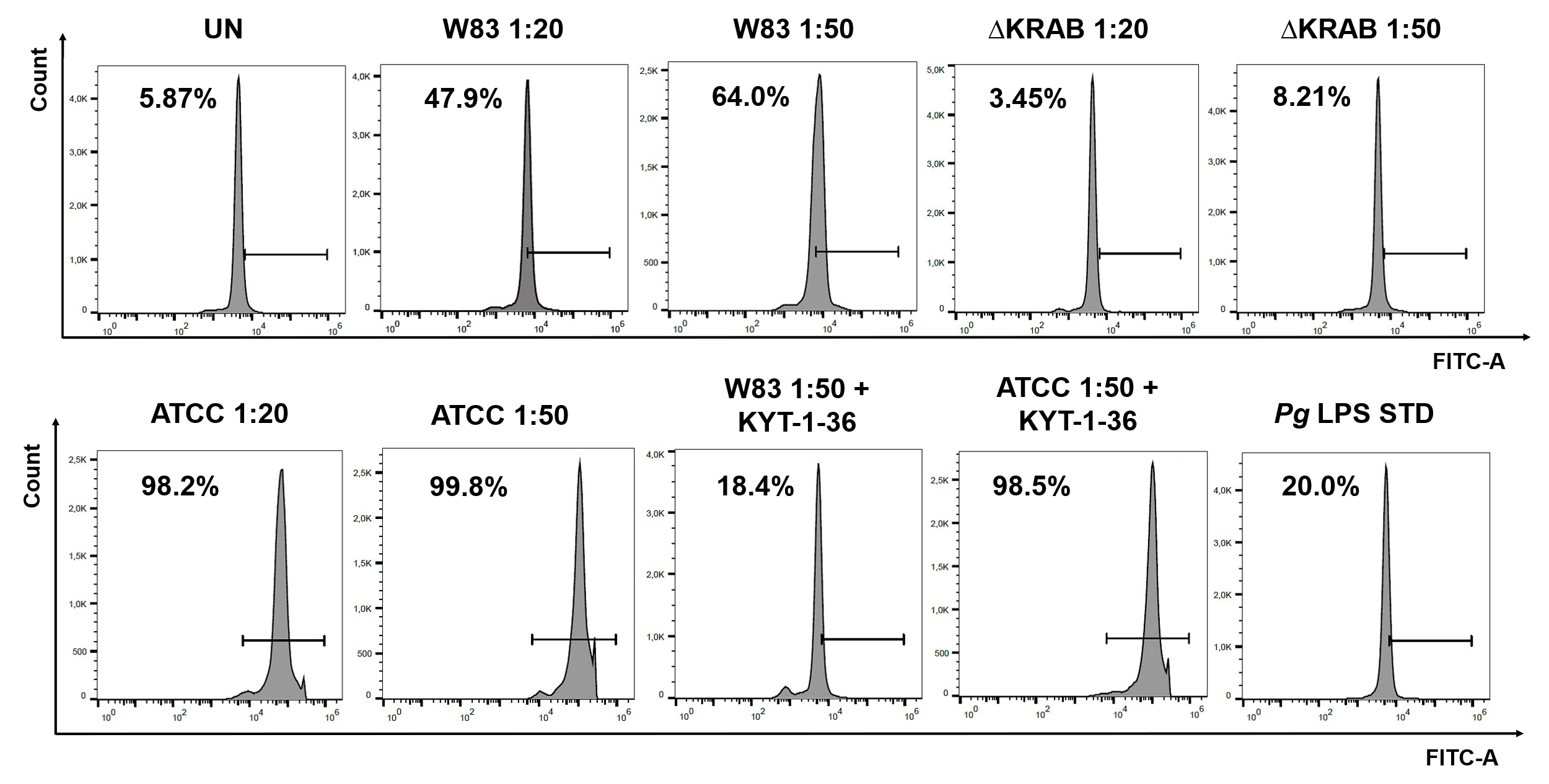


**Supplementary Figure S7. Gingipains induce strong ROS production by primary human neutrophils.** Primary human neutrophils were challenged with W83 or ATCC 33277, or the gingipain mutant in the absence and presence of gingipain inhibitors KYT-1 and KYT-36 [both at 1 μM]. Bacteria were used at MOI 20 and 50. *P. gingivalis*-derived LPS Standard (*Pg* LPS STD) served as a positive control [1 µg/ml]. Untreated (UN) cells were used as a negative control. Production of ROS by primary human neutrophils was analyzed by staining with DCFH_2_-DA for 2 h, followed by measurement of fluorescence emitted by oxidized DCF+.


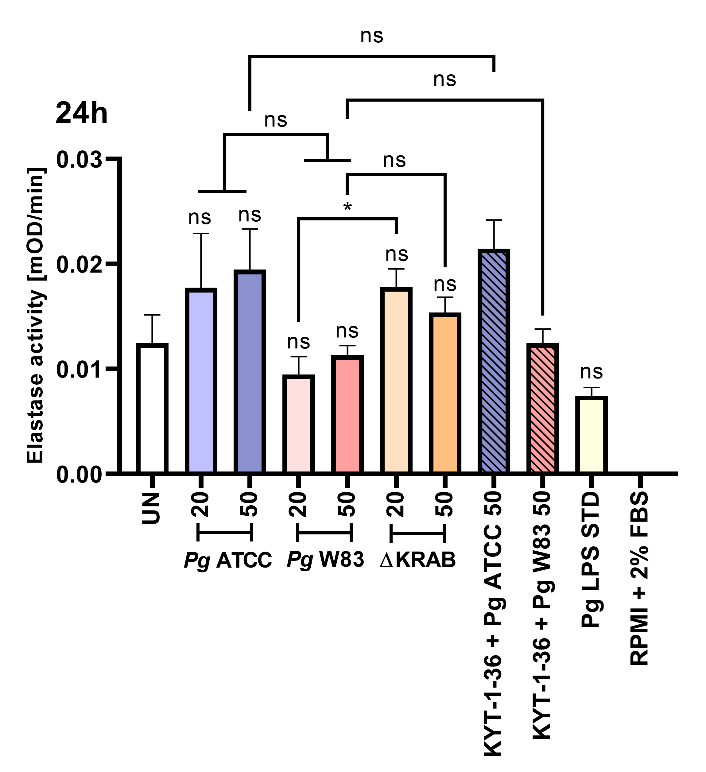


**Supplementary Figure S8. Gingipains affect the activity of human neutrophil elastase.** Primary human neutrophils were challenged with wild-type *P. gingivalis* (W83 and ATCC 33277) or the mutant devoid of gingipain activity (∆KRAB) in the absence and presence of gingipain inhibitors KYT-1 and KYT-36 [1 μM], the supernatants were collected, and hNE activity was measured in the presence of chromogenic substrate after 24 h. Data are presented as the mean ± SEM of 3 independent experiments, and compared to UN cells. *p < 0.05, ns = not significant (One-way ANOVA followed by the Dunnett post-hoc test, One-way ANOVA followed by the Bonferroni post-hoc test and Unpaired *t*-test). hNE activity levels were measured in duplicate. UN cells and cell culture medium alone (RPMI with 2% FBS) were used as negative controls for the experiments.

**
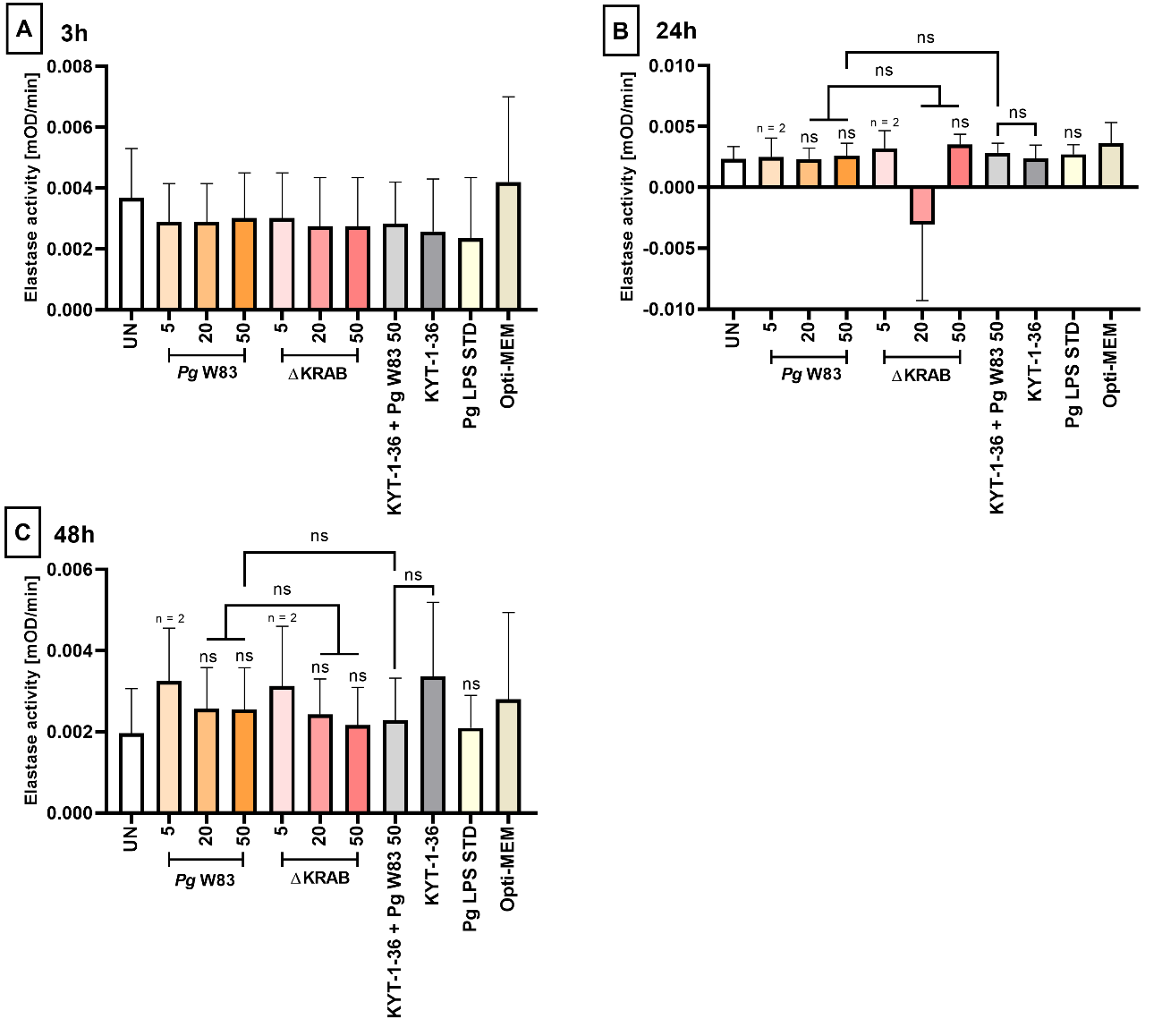
**

**Supplementary Figure S9. *P. gingivalis* does not affect the elastase activity of murine HoxB8 neutrophils.** Murine HoxB8 neutrophils (representative WT1 neutrophil line) were stimulated with the WT-W83 or the ∆KRAB mutant at MOI 5, 20, or 50, and in the presence/absence of gingipain inhibitors KYT-1 and KYT-36 [both at 1 µM]. A *P. gingivalis*-derived LPS Standard (*Pg* LPS STD) served as a positive control [1 µg/ml]. The supernatants were collected, and neutrophil elastase (NE) activity was measured in the presence of chromogenic substrate after **(A)** 3, **(B)** 24, and **(C)** 48 h. Data are presented as the mean ± SEM of **(A)** 2, **(B, C)** 2-3 independent experiments and compared to untreated (UN) cells. ns = not significant (One-way ANOVA followed by the Dunnett post-hoc test, Kruskal-Wallis test followed by the Dunn post-hoc test, One-way ANOVA followed by the Bonferroni post-hoc test and Unpaired *t*-test). NE activity levels were measured in duplicate. UN cells and cell culture medium alone (Opti-MEM) were used as negative controls for the experiments.

**2. Supplementary methods**

**HoxB8 macrophage derivation and treatment**

HoxB8 macrophage progenitors were grown and differentiated in GM-CSF containing medium (1% of genetically modified B16 cell line, as described before (52)). Survival was analyzed using flow cytometry after Annexin V-BV421 and propidium iodide (PI) staining. Double negative cells (AnnexinV^-^PI^-^) were calculated as viable.

**Bacterial cultures of *P. gingivalis* fimbriae mutants**

*P. gingivalis* wild-type strain ATCC 33277 and two derived mutants lacking fimbriae proteins *Δmfa1* and *ΔfimA* were grown on blood agar plates. The mutants were grown on agar plates supplemented with erythromycin (5 μg/ml). The construction of *Δmfa1* and *ΔfimA* was described, respectively (58, 59).
